# Development and quality characteristics of shelf‐stable soy‐agushie: a residual by‐product of soymilk production

**DOI:** 10.1002/fsn3.292

**Published:** 2015-10-09

**Authors:** Christina A. Nti, Wisdom A. Plahar, Nana T. Annan

**Affiliations:** ^1^ Department of Family and Consumer SciencesUniversity of GhanaLegonGhana; ^2^ CSIR‐Food Research InstituteBox M.20AccraGhana

**Keywords:** Product development, quality characteristics, soy‐agushie, soymilk by‐product

## Abstract

A process was developed for the production of a high‐protein food ingredient, *soy‐agushie*, from the residual by‐product of soymilk production. The product, with a moisture content of about 6%, was evaluated for its quality characteristics and performance in traditional dishes. The protein content was about 26% with similar amino acids content as that of the whole soybean. Lysine remained high in the dehydrated product (6.57 g/16 g N). While over 60% of the original B vitamins content in the beans was extracted with the milk, high proportions of the minerals were found to be retained in the residual by‐product. The process adequately reduced the trypsin inhibitor levels in the beans from 25 to 1.5 mg/g. High sensory scores were obtained for recipes developed with soy‐agushie in traditional dishes. The scope of utilization of the soy‐agushie could be widened to include several traditional foods and bakery products for maximum nutritional benefits.

## Introduction

In many parts of Africa, soybean has been identified as a suitable protein‐rich crop that could help improve the economic and nutritional status of the general population through increased production and utilization. It is for this reason that several projects were initiated in the early 2000s with the objective to promote the production and utilization of soybeans in the West African sub‐region through the development of home‐level and small‐scale processing technologies for soy‐based foods for rural and urban populations (IDRC Soybean Utilization Project document, 1994). The processing technologies are aimed at developing new food products and improve the quality of local foods by fortifying them with soybeans, taking into account country‐specific and within country preferences. Promotion of the production and consumption of soymilk in Ghana is one of the main activities of the soybean utilization research groups in Ghana, Nigeria, and Cote D'Ivoire in one such collaborative project.

Technologies have been developed and extended in past research work for the preparation of soymilk both at the household level and the small‐scale enterprise level (Plahar and Annan 1994). One major by‐product of soymilk production is the residue after extraction of the milk. Food uses of the wet residue have not been developed. As a result, producers end up giving it to free range domestic animals. The apparent similarities in the physical characteristics of the soy residue and ground melon seed (*agushie*) used extensively for various food preparations in West Africa formed the basis for the present investigation aimed at developing an acceptable substitute for agushie. Agushie is used in countries in the West African sub‐region for the preparation of diverse soups and stews which form a major part of the diets of the people. It is, however, relatively expensive and not always readily available. The method of preparation of the mashed agushie is also tedious as it has to be dehulled manually and ground on a stone or mill. It is envisaged that the development of a stable and ready‐to‐use product from the residual by‐product of soymilk production will not only be nutritionally advantageous, but will also add economic value to the utilization of soybean for milk production.

The purpose of the study was therefore to develop an acceptable agushie substitute from the residual by‐product of soymilk production and to characterize the product in terms of its nutritional, sensory, and physicochemical properties. Acceptability of the soy‐agushie as a substitute for the melon seed was also assessed in a few traditional stews and sauces.

## Materials and Methods

### Materials

Samples of soybeans used in this study were the *Salintuya* variety obtained from the CSIR‐Crops Research Institute, Ghana. All chemicals used were of reagent grade obtained from the Sigma Chemical Co. (St. Louis, MI).

### Product development

The standard procedure reported by Plahar and Annan (1994) was followed in the preparation of soymilk samples. For each batch production, cleaned soybeans were soaked in potable water (1:5 w/v) for 30 min. The water was drained and the soaked beans dropped into boiling water (1:10 dry bean weight to water ratio) and allowed to boil for 10 min with 0.2% salt added. A slurry was prepared by grinding the blanched beans for 3 min in a blender using all of the blanch water. The slurry was stirred well and filtered hot through muslin cloth, squeezing to remove as much filtrate as possible. The extract obtained was boiled for 20 min to obtain the milk. The wet residual by‐product was dried for about 6 h in a mechanical dryer (Apex, Royce Ross Ltd., Construction Ltd. Chemical Eng., Soho Sq. London) maintained at 70–80°C to obtain the relatively shelf‐stable dehydrated meal with a moisture content of 3.6%. The dehydrated meal was milled using a hammer mill (Jacobson Machine Works Inc., Minneapolis, MN) into grits and sealed in double‐laminated sealable polyethylene bags as the shelf‐stable soy‐agushie. Color of the final product was one main physical characteristic used to determine the optimal dehydration parameters. The color of the samples was measured in triplicate using the L*, a*, and b* color space (CIE LAB space) with Colorimeter CR‐200 (Minolta, Model CR310, Minolta Camera Company Ltd., Osaka, Japan). The L* value indicates lightness, where L* = 0 is completely black and L* = 100 is completely white. The a* values represent red to green with positive a* and negative a* depicting red and green, respectively. The b* values on the other hand represent yellow to blue, with positive b* representing yellow and negative b* representing blue. The meter was calibrated with a white tile (L* = 97.63, a* = −0.48, and b* = +2.12). ∆E values were calculated to indicate the extent of deviation of color of samples from the standard tile color used. ∆E is calculated as the square root of the sum of the squared deviations of L*, a*, and b* values, that is, $\Delta E=\sqrt{\left[\Delta L^{2}+\Delta a^{2}+\Delta b^{2}\right]}$ (Morrison and Laignelet 1983; Oduro‐Yeboah et al. 2010).

### Recipe formulation and sensory evaluation

Recipes were developed with the soy‐agushie as a replacement for the melon seed in common traditional stews, sauces, and soups including gravies, ordinary stews, garden egg stew, palaver sauce, vegetable soup, and groundnut soup. In the recipe development with the soy‐agushie, the technical development processes for optimization and prototype refining by sensory techniques were applied to arrive at the final ingredient composition and preparation of each recipe. Sensory characteristics of the products were evaluated by a 10‐member trained panel of the CSIR‐Food Research Institute, Ghana. The quantitative descriptive sensory analysis as described by Johnson et al. (1988) was used to assess the color of the products. To determine the relative intensity of the beany flavor in the samples, the category scaling method of Larmond (1977) was used. Samples were served at room temperature in porcelain plates and judges were asked to evaluate the beany flavor by checking the appropriate terms on a graduated scale with anchor words. The triangle difference test (Larmond 1977) was used to compare traditional agushie (lemon seed) products and the soy substitutes.

### Analytical procedures

The following analytical procedures were used for a comprehensive quality assessment of the wet and the dehydrated meal products as well as the milk extract. Product quality factors determined include the proximate composition, minerals (sodium, potassium, calcium, magnesium, phosphorus, iron, manganese, zinc, and copper), vitamins (total pyridoxamine, riboflavin, and biotin), selected disaccharides (fructose, sucrose, and maltose), amino acids, and TIA (trypsin inhibitor activity).

Moisture (AOAC 925.10), protein (AOAC 984.13), fat (AOAC 920.39), and ash (AOAC 923.03) were determined by the AOAC (2000) standard methods, while carbohydrates were calculated by difference, and energy values were obtained using the Atwater factors 3.47, 8.37 and 4.00 for protein, fat, and carbohydrates, respectively (Eyeson and Ankrah 1975). Iron, calcium, and phosphorous were determined by AACC (2000) bipyridyl colorimetric, permanganate titration, and molybdenum methods, respectively, while sodium, potassium, calcium, magnesium, manganese, zinc, and copper were determined using Atomic Absorption Spectrophotometric method with the PerkinElmer atomic absorption spectrophotometer No.3030, (AACC, 1999). The ground samples were ashed and dissolved in a mixture of 0.5 mL nitric acid and 1 mL hydrochloric acid, heated, and diluted after cooling. Measurements were taken against standards.

A modification of the HPLC (high‐performance liquid chromatography) method described by Aslam et al. (2008) and Ekinci and Kadakal (2005) was used for the determination of riboflavin (vitamin B_2_), pyridoxine (vitamin B_6_), and biotin (vitamin B_7_), with simultaneous UV and fluorescence detection. Two to seven gram samples were weighed into a 100 mL volumetric flask and hydrolyzed for 30–60 min with 50 mL water + 1 mL 25% HCl at 70°C in a water bath. After cooling to 40°C the sample solution was buffered with 5 mL 2.5 N sodium acetate solution and incubated overnight with 1 mL freshly prepared enzyme solution (4 mg/mL phosphatase and 15 mg/mL Taka‐Diastase) at 37°C. The solution was cooled, made up to the mark with deionized water, and filtered. The filtrate was further washed over a C18‐SPE ion exchange column and the eluent was measured by HPLC.

The HPLC technique of Müller and Siepe (1980) was used for the determination of fructose, glucose, galactose, sucrose, maltose, and lactose contents of the samples. The watery solution of each sample was clarified with Carrez solution (K_4_(Fe(CN)_6_).3H_2_O and ZnSO_4_.7H_2_O) and cleaned on an SPE column (Waters Sep‐Pak No. 51910). Methyl‐*α*‐d‐glucopyranoside was used as internal standard. The components were then separated on an HPLC (column Nucleosil NH_2_, 5 mm, 250 × 4 mm with a 30 × 4 mm fore column), using a refractive index detector. HPLC conditions were flow rate = 1 mL/min, sample volume = 20 mL, column temperature = 30°C, refractive index temperature = 35°C, eluent = acetonitrile:water ratio of 70:20 w/w.

Amino acid composition of samples was determined in triplicate by digestion under vacuum with 6 N HCl in sealed ampoules at 110°C for 22 h. The hydrolysates were derivatized and analyzed for amino acids on a Waters HPLC system controlled by Millenium 2010 software (Waters Div., Millipore Corp., Milford, MA). Cystine was determined as cysteic acid by performic acid oxidation (Hirs 1967) as described by Albert et al. (2008). The colorimetric technique of Opienska‐Blauth et al. (1963) was used for the determination of tryptophan in extracts prepared by the method of Subramanian et al. (1970).

Trypsin inhibitor activity was determined by the method of Hammerstrand et al. (1981). One gram portions of the samples were extracted by soaking overnight at 4°C in 50 mL 0.01 NaOH (pH was adjusted to 8.4–10.0). The suspensions were diluted so that 2 mL of the sample extract inhibited 40–60% of standard trypsin used in the analysis. For the analysis on inhibition of trypsin, synthetic BAPNA (benzoyl dl arginine‐*p*‐nitro anilide) was used as substrate. Residual enzyme activities were determined in systems containing 2 mL aliquots of the sample extracts by measuring the absorbance at 410 nm. TIA in terms of milligrams pure trypsin per gram sample was calculated as

$$\text{TIA}\;=\;\frac{2.632\;\times \;D\times A_{1}}{S}$$

where A_1_ = change in absorbance due to trypsin inhibition per mL diluted sample extract, D = dilution factor, and S = weight of sample (g).

### Statistical analysis

Statistical significance of observed differences among means of experimental results were evaluated by ANOVA (analysis of variance) followed by pairwise comparison of means (Steel and Torie 1981). Significance was accepted at *P* < 0.05.

## Results and Discussion

### Dehydration of soy‐agushie samples

Using the commercial Apex dryer (Apex, Royce Ross Ltd., Construction Ltd. Chemical Eng., Soho Sq. London) the optimal dehydration temperature range for the soy‐residue samples was found to be between 70°C and 80°C. The desired physical and sensory characteristics were obtained at this temperature while higher temperatures presented problems of burnt flavor and lower temperatures prolonged the drying rate unduly. There was no detectable beany flavor in the dried meal, and the color of the product was creamy off‐white with L*, a*, and b* values of 82.64 ± 0.35, −1.62 ± 0.08, and 12.51 ± 0.39, respectively, and a ΔE value of 18.27. The ΔE value indicates the extent of deviation of color of the product from the standard tile color used (L* = 97.63, a* = −0.48, and b* = +2.12). In other studies ΔE values obtained using the same standard tile for similar products were 15.61 for yam bean flour obtained by drying at 55°C for 5½ h (Buckman et al. 2015), and 29.98 for orange flesh sweet potato flour obtained by drying in a solar tent dryer at 40–50°C for 6 days (Bonsi et al. 2014). Plahar et al. (1983) recommended a dehydration temperature of 62°C for unfortified fermented maize dough to produce dehydrated maize meal with the desired color characteristics.

### Chemical characteristics of soy‐agushie samples

The proximate composition and trypsin inhibitor activities of soymilk extract and the residual by‐product, soy‐agushie samples are given in Table 1. On dry weight basis, the protein and fat contents of the soy‐residue were significantly (*P* < 0.05) higher than in the soymilk. A large proportion of the protein and fat contents of the original grain were not extracted into the milk, but remained in the residue making the latter highly nutritious. The milk extraction procedure could extract only a small proportion of the protein and fat from the raw grains, with the dried residue containing over 70% and 56%, respectively, of the original content of these nutrients. A similar situation was observed in the ash content of the extract and the residual by‐product. The soy‐agushie prepared from the residue therefore had nutritional quality quite close to that of the original grains used. The composition of the undehydrated fresh wet residue, when converted to dry weight basis is quite similar to that of the dehydrated meal.

**Table 1:** Proximate composition and trypsin inhibitor activity of soymilk and the residual by‐product, soy‐agushie samples^a^

Component	Whole raw soybean	Soymilk sample	Fresh (wet) soy‐agushie	Dehydrated soy‐agushie
Moisture (%)	9.54 ± 0.24	88.62 ± 0.72	59.12 ± 0.51	5.73 ± 0.70
Protein (%)	37.41 ± 0.64	2.53 ± 0.91	10.91 ± 1.71	26.40 ± 1.13
Fat (%)	18.72 ± 1.14	1.58 ± 0.66	4.73 ± 0.44	10.50 ± 0.62
Ash (%)	6.12 ± 0.32	0.60 ± 0.15	1.01 ± 0.34	3.72 ± 0.53
Carbohydrates (%)	28.21 ± 1.33	6.67 ± 1.34	24.23 ± 1.87	53.66 ± 1.56
Energy (kcal)	399.34 ± 4.32	48.68 ± 2.90	174.37 ± 5.07	394.13 ± 4.17
TIA (mg/g sample)^b^	25.36 ± 1.72	0.31 ± 0.06	1.82 ± 0.30	1.53 ± 0.44

a Values are mean ± standard deviation of triplicate determinations expressed on as‐is basis.

b Trypsin inhibitor activity.

In terms of antinutritional factors, the heat treatment was found adequate in drastically reducing the trypsin inhibitor levels in soy‐agushie from an original value of 25 mg CE/g sample to minimal levels of <2 mg CE/g soy‐agushie sample, and <0.5 mg CE/g soymilk sample. This situation is quite desirable to guarantee no adverse trypsin inhibitory effects in cases where minimal heat treatments will be applied in culinary preparations of dishes containing soy‐agushie. The TIA in the dehydrated soy‐agushie meal was found to be lower than that in the wet meal when considered on dry weight basis (Table 1). The dehydration process helped to further destroy the residual activity in the dried product. In other studies, Plahar and Annan (1994) observed a reduction in TIA from 25 to 0.3 mg/g sample after boiling soaked soybeans for 20 min. Such final levels of TIA are considered safe for maximum nutritional benefits.

### Minerals, vitamins, and sugar content of soy‐agushie samples

Table 2 gives the concentrations of selected minerals, vitamins, and sugars in soymilk extract and the soy‐agushie samples compared to the content in the original raw soybean. It was observed that most of the minerals determined were in greater concentrations in the final dehydrated soy‐agushie product than in the raw material. The normal preparation of soymilk involves addition of sodium chloride in the form of crude table salt. The normal crude salt used usually contains small amount of mineral contaminants, and this should account for the greater concentrations of some of the minerals in the product than in the beans. In general, however, large proportions of the mineral content of the raw beans were found to be retained in the residual by‐product during soymilk extraction. On dry weight basis, the amount of minerals extracted with the milk was found to be at most 22% of the unextracted portion in the residue. In most cases, the extracted portions were as low as 4% of the unextracted portions. The minerals in the greatest concentrations in the soy‐agushie were potassium, calcium, magnesium, and phosphorus. Minor mineral components were sodium, iron, manganese, zinc, and copper (Table 2).

**Table 2:** Minerals, vitamins, and sugar content of raw soybean, soymilk, and soy‐agushie samples^a^

Component	Whole raw soybean	Soymilk sample	Dehydrated soy‐agushie
Minerals (mg/100 g)			
Sodium	1.6 ± 0.28^c^	2.5 ± 0.34^b^	12.2 ± 0.68^a^
Potassium	1,269 ± 8.10^a^	206.0 ± 5.02^c^	945.0 ± 6.75^b^
Calcium	268.0 ± 2.60^b^	18.5 ± 1.25^c^	347.0 ± 3.24^a^
Magnesium	182.0 ± 4.30^a^	22.0 ± 0.94^c^	151.0 ± 1.77^b^
Phosphorus	617.7 ± 3.60^a^	60.3 ± 1.81^b^	641.1 ± 2.89^a^
Iron	9.1 ± 0.81^a^	0.5 ± 0.08^b^	9.5 ± 1.23^a^
Manganese	1.6 ± 0.31^a^	0.1 ± 0.02^b^	2.4 ± 0.22^a^
Zinc	4.0 ± 0.58^a^	0.3 ± 0.04^b^	4.5 ± 0.38^a^
Copper	1.4 ± 0.19^a^	0.2 ± 0.06^b^	1.5 ± 0.42^a^
Vitamins (mg/100 g)			
Pyridoxin—HCl	0.36 ± 0.07^b^	0.53 ± 0.08^a^	0.17 ± 0.08^c^
Pyridoxamine	0.19 ± 0.04^a^	0.13 ± 0.03^a^	0.04 ± 0.01^b^
Total B_6_	0.55 ± 0.08^a^	0.66 ± 0.17^a^	0.21 ± 0.08^b^
Riboflavin	0.14 ± 0.02^a^	0.02 ± 0.01^c^	0.06 ± 0.01^b^
Biotin	0.03 ± 0.01^a^	–	0.02 ± 0.00^a^
Sugars (g/100 g)			
Fructose	–	0.12 ± 0.09^a^	–
Sucrose	3.61 ± 0.21^a^	1.07 ± 0.18^b^	1.50 ± 0.17^b^
Maltose	0.44 ± 0.05^a^	0.15 ± 0.02^b^	0.03 ± 0.01^b^

a Values are mean ± SD of triplicate determinations expressed on dry weight basis. Means with different superscript letter are significantly different (p<0.05).

Contrary to the observations made on the minerals, relatively large proportions of the vitamins were extracted with the milk leaving significantly (*P* < 0.05) lower concentrations in the residue. The vitamins determined were water soluble, and were more likely to be concentrated in the aqueous milk phase. In spite of this, the soy‐agushie was found to contain significant levels of the B vitamins. Similarly, most of the sugars were extracted into the milk. Although fructose was not determined in the soybean, about 0.12% was observed in the milk extract. This could have been produced during the process. Earlier reports by Hou et al. (2009) mentioned about 4.49 mg/g sucrose, 0.12 mg/g fructose, and 0.18 mg/g glucose in soybeans.

### Amino acid composition of soymilk and soy‐agushie

Table 3 gives the amino acid composition of raw soybeans, soymilk extract, and the wet and dehydrated residual by‐products. The amino acid composition of the soymilk extract was similar to that of the raw soybean. However, the residual by‐product was found to have higher concentrations of most of the amino acids than in the original beans. Lysine remained high in both the wet and the dehydrated products, as well as in the milk ranging between 6.47 and 6.57 g/16 g N in the meals and 6.66 g/16 g N in the milk. Tryptophan content was also high in the product. The dehydration temperatures used were also mild enough to prevent loss of available essential amino acids in the process. Considering the fact that traditional cereal‐based weaning foods in Ghana are grossly inadequate in these two amino acids, and hence their low nutritive value, the soy‐agushie could serve as a fortifying material in these traditional weaning foods for improved amino acids pattern and general protein content and quality.

**Table 3:** Amino acid composition (g/16 g N) of raw soybean, soymilk, and soy‐agushie (wet and dried) samples^a^

Component	Whole raw soybean	Soymilk sample	Fresh (wet) soy‐agushie	Dehydrated soy‐agushie
Aspartic acid	10.80 ± 0.51	11.54 ± 0.42	16.11 ± 0.28	16.09 ± 0.35
Threonine	3.83 ± 0.30	4.11 ± 0.38	5.47 ± 0.39	5.72 ± 0.21
Serine	5.16 ± 0.15	5.24 ± 0.24	7.47 ± 0.40	7.75 ± 0.38
Glutamic acid	17.09 ± 0.13	19.40 ± 0.47	25.47 ± 0.21	25.26 ± 0.32
Proline	5.30 ± 0.20	5.51 ± 0.18	8.21 ± 0.41	8.09 ± 0.44
Glycine	4.02 ± 0.32	4.36 ± 0.19	6.00 ± 0.50	6.09 ± 0.21
Alanine	3.97 ± 0.11	4.14 ± 0.30	6.00 ± 0.22	6.27 ± 0.20
Cystine	1.40 ± 0.10	1.68 ± 0.08	2.00 ± 0.10	1.97 ± 0.12
Valine	4.14 ± 0.20	3.92 ± 0.18	6.63 ± 0.19	6.70 ± 0.23
Methionine	0.98 ± 0.13	1.33 ± 0.22	1.89 ± 0.12	1.38 ± 0.28
Isoleucine	4.09 ± 0.23	3.97 ± 0.27	6.63 ± 0.51	6.55 ± 0.48
Leucine	6.99 ± 0.57	6.75 ± 0.45	10.84 ± 0.55	10.92 ± 0.54
Tyrosine	3.05 ± 0.17	3.53 ± 0.22	5.16 ± 0.36	4.55 ± 0.16
Phenylalanine	4.79 ± 0.27	4.57 ± 0.23	7.68 ± 0.26	7.51 ± 0.18
Lysine	6.34 ± 0.41	6.66 ± 0.12	6.47 ± 0.21	6.57 ± 0.20
Histidine	2.62 ± 0.08	2.82 ± 0.11	3.79 ± 0.17	3.94 ± 0.10
Arginine	6.78 ± 0.27	7.63 ± 0.18	10.63 ± 0.30	10.06 ± 0.26
Tryptophan	1.43 ± 0.60	1.30 ± 0.08	2.00 ± 0.41	2.06 ± 0.13

a Values are mean ± SD of triplicate determinations expressed on dry weight basis.

Previous studies investigated the effects of fortification of traditional cereal‐based weaning foods with soy flour. The results showed significant improvements in the protein content and quality of the traditional foods by virtue of the high‐protein content of the soy flour and by the mutual complementation of the limiting amino acids (Plahar et al. 1983, 1997). The protein content of the soy‐agushie developed in the present study was comparable to that of the soy flour, with a better amino acids pattern in terms of high concentrations of lysine, tryptophan, and the sulfur amino acids. These qualities would make the soy‐agushie an equally good, if not a better material for the development of high‐protein weaning foods based on traditional cereal‐based weaning foods. Other quality characteristics of blends with respect to their biochemical, hematological, histopathological, and nutritional implications would, however, have to be established through animal studies.

### Soy‐agushie recipes

Six recipes were developed with soy‐agushie as a substitute for the melon seed, based on traditional dishes. The ingredient composition and preparation methods were arrived at after several tests for minimal detectable differences between test samples and traditional controls, as well as maximum consumer acceptability of sensory attributes. The mean sensory scores for the final recipes are shown in Table 4. For all the sensory attributes, mean scores were similar for both traditional agushie products and products with soy‐agushie as substitute. Consumer overall acceptability ranged between 7.0 and 7.9 which indicates moderate to very much liking of the products. Mean scores for taste ranged from 6.4 (like slightly) for the groundnut soup to 7.8 (like very much) for the palaver sauce. Aroma scores ranged between 6.9 (like moderately) for vegetable soup and 8.1 (like very much) for palaver sauce. Similarly, texture/consistency scores also indicate high acceptability with scores ranging between 7.3 (like moderately) for vegetable soup and 8.4 (like very much) for the soy‐agushie gravy. In general, soy‐agushie was more acceptable in stews and sauces than in soups for which the traditional agushie also received relatively low scores.

**Table 4:** Mean sensory scores for recipes developed with soy‐agushie in selected traditional dishes^a^

Product	Sensory attributes			
	Taste	Aroma	Texture/consistency	Overall acceptability
Gravy				
Traditional	7.1 ± 0.73	7.4 ± 1.24	8.3 ± 1.02	7.8 ± 0.74
Soy‐agushie	6.8 ± 1.10	7.5 ± 0.95	8.4 ± 1.23	7.5 ± 0.68
Tomato stew				
Traditional	7.0 ± 0.75	7.2 ± 0.92	8.1 ± 1.30	7.6 ± 1.03
Soy‐agushie	6.7 ± 0.46	7.3 ± 1.19	7.8 ± 1.21	7.4 ± 1.24
Garden egg stew				
Traditional	7.6 ± 1.03	7.8 ± 0.64	7.9 ± 0.92	7.7 ± 0.92
Soy‐agushie	7.7 ± 1.18	7.4 ± 0.74	8.1 ± 1.24	7.6 ± 0.64
Palaver sauce				
Traditional	8.2 ± 1.12	8.0 ± 1.19	7.9 ± 0.99	8.0 ± 1.24
Soy‐agushie	7.8 ± 1.06	8.1 ± 1.20	7.6 ± 0.75	7.9 ± 1.17
Vegetable soup				
Traditional	6.6 ± 0.75	6.8 ± 0.46	7.5 ± 1.04	7.2 ± 0.74
Soy‐agushie	6.8 ± 0.70	6.9 ± 0.64	7.3 ± 1.21	7.0 ± 0.63
Groundnut soup				
Traditional	6.2 ± 0.48	7.5 ± 1.19	8.0 ± 1.03	7.3 ± 0.87
Soy‐agushie	6.4 ± 0.74	7.8 ± 1.23	7.5 ± 0.98	7.1 ± 1.06

a Interpretation of scores: 9 = like extremely; 8 = like very much; 7 = like moderately; 6 = like slightly; 5 = neither like nor dislike; 4 = dislike slightly; 3 = dislike moderately; 2 = dislike very much; 1 = dislike extremely.

## Conclusion

The process developed in the study effectively produced a nutritious shelf‐stable soy‐agushie product that could be effectively used as a substitute for agushie in West African traditional dishes. The product contains lysine and tryptophan in concentrations that can contribute significantly to improving the amino acid pattern of cereal–legume blends. The milk extraction procedure was able to extract only small proportions of the protein and fat from the raw seeds, leaving the dried residue soy‐agushie with about 26.4% protein and 10.5% fat content. About 50% of the B vitamins in the original content of the beans were extracted with the milk, while very high proportions of the minerals determined were found to be retained in the residue. The heat treatment adequately reduced the trypsin inhibitor levels in the soy‐agushie from 25 mg/g to minimal levels of 1.5 mg/g. Recipes developed with the soy‐agushie as replacement for melon seed in traditional dishes had sensory scores in the same category of degree of likeness as the traditional dishes. The scope of utilization of the soy‐agushie developed could be widened to include several traditional foods, bakery products, and weaning foods. There is however the need to develop appropriate recipes and large‐scale consumer acceptability tests.

## Conflict of Interest

None declared.
